# Linear and Continuous Flavivirus Epitopes From Naturally Infected Humans

**DOI:** 10.3389/fcimb.2021.710551

**Published:** 2021-08-12

**Authors:** Marcilio Jorge Fumagalli, Luiz Tadeu Moraes Figueiredo, Victor Hugo Aquino

**Affiliations:** ^1^Virology Research Center, Medical School of Ribeirão Preto, University of São Paulo, Ribeirão Preto, Brazil; ^2^Laboratory of Virology, Department of Clinical Analyses, Toxicology and Food Sciences, Faculty of Pharmaceutical Sciences of Ribeirão Preto, University of São Paulo, Ribeirão Preto, Brazil

**Keywords:** linear epitopes, immunoreactive peptides, flavivirus, human antibodies, arbovirus

## Abstract

This manuscript is an up-to-date review of experimentally validated linear and continuous epitopes identified from arbovirus members of the *Flavivirus* genus. We summarized 153 immunoreactive peptides from the Dengue virus, Zika virus, Japanese encephalitis virus, West Nile virus, and tick-borne encephalitis virus described in studies published from 1989 to 2020. We included peptides from structural (envelope, capsid, and pre-membrane) and nonstructural (Ns1–5) viral proteins that demonstrated relevant immunoreactivity with antibodies from naturally infected or vaccinated humans. We included peptides that demonstrated relevant reactivity features, such as indicators of disease severity related to immunological or immunopathological outcomes, differential or group diagnostic markers, immunotherapy candidates, and potential for vaccine formulation. The majority of immunoreactive peptides were described for DENV probably due to its long-lasting impact on human health and the lack of efficient vaccines and therapeutic methods. Immune landscape data regarding linear immunoreactive and continuous flavivirus peptides are still scarce, and a complete and more detailed map remains to be elucidated. Therefore, this review provides valuable data for those investigating the antibody response against flavivirus infection.

## Introduction

The *Flavivirus* genus belongs to *Flaviviridae* family and currently comprises 53 recognized viral species, many of which are important human pathogens (Simmonds et al., 2017). Most flaviviruses are arthropod-borne viruses with a cycle that involves transmission between hematophagous arthropods and vertebrate hosts, which in many cases results in a disease-related infection (D.J. et al., 2007). The Dengue virus (DENV), Zika virus (ZIKV), West Nile virus (WNV), Japanese Encephalitis virus (JEV), and tick-borne encephalitis virus (TBEV) are among the most prominent representatives of arboviruses.

The worldwide spread of flaviviruses in the last decades has been remarkable. The DENV alone accounts for approximately 96 million human cases each year and exposes more than 3.9 billion people to the risk of infection, representing a large economic and public health concern (Bhatt et al., 2013). The ZIKV has recently emerged as a global threat, demonstrating the ability to spread explosively; it affects more than 85 countries and territories in Africa, America, Southeast, Asia and the Western Pacific region, causing considerable morbidity related to autoimmune syndromes and congenital diseases (2019; Pierson and Diamond, 2018). The WNV is one of the most widely distributed arboviruses in the world and is commonly found in Africa, Europe, the Middle East, North America, and West Asia, where it causes neurological disease and death in humans and animals (Petersen et al., 2013). The JEV is responsible for encephalitis in humans and circulates in temperate, subtropical, and tropical regions of Asia, northern Australia, and more recently in Africa, exposing more than 2 billion people to the risk of infection (Filgueira and Lannes, 2019). The TBEV is an important viral infection of the central nervous system (CNS) that affects many countries in Europe and Asia, causing a range of disease symptoms that can progress from mild to severe meningoencephalitis (Bogovic and Strle, 2015). Thus, the emergence and re-emergence of flaviviruses represent a continuous global threat that can unexpectedly emerge in human populations and cause a diverse spectrum of diseases.

## Flavivirus Proteins and Replication

Flaviviruses are small-enveloped viruses (approximately 50 nm in diameter) with a single linear positive-sense RNA genome of approximately 11 kbp. The genomic RNA presents a single open-reading frame (ORF) that is translated into a single polyprotein precursor of approximately 3,400 amino acids. This precursor polyprotein is co-translationally processed by host and virus-specific proteases to yield three structural proteins (capsid (C), pre-membrane (prM), and envelope (E)), which are responsible for assembling the viral particle, and seven nonstructural proteins (Ns1, Ns2A, Ns2B, Ns3, N4A, Ns4B, and Ns5), which are primarily involved in viral replication (Mukhopadhyay et al., 2005).

The structural proteins are responsible to compose the viral particle, mediate protection and interaction it susceptible cells. The viral particle comprises an electron-dense core that contains a single copy of the single-stranded genomic RNA molecule complexed with multiple copies of the capsid protein. The capsid is a small helical protein that interacts with viral RNA *via* its basic residues and with the viral membrane *via* its hydrophobic cleft (Ma et al., 2004). It is composed of four alpha-helical structures that form antiparallel homodimers, with one face of the capsid dimer being highly charged and the opposite face containing a hydrophobic cleft (Fu et al., 1992). The viral core is surrounded by a lipid bilayer where transmembrane viral proteins are inserted to form the viral shell, which consists of 180 copies of each of the envelope and prM proteins (Byk and Gamarnik, 2016). The prM protein is incorporated into the viral envelope in heterotrimeric prM–E spikes that protect the interaction of the fusion loop on the envelope with the host membrane during viral egress, preventing the fusion of recently synthesized virions (Zhang et al., 2003). The envelope is a glycosylated protein divided into three structural domains (DI, DII, and DIII), a helical stem, and a transmembrane domain. On the viral particle surface, the envelope proteins are arranged in antiparallel dimers embedded on the viral membrane (Zhang et al., 2017).

The nonstructural proteins are involved in viral replication and modulation of the host antiviral response; however, they remain poorly characterized to date. The Ns1 is a large glycoprotein, which is presented as homodimers on the host cellular membrane or as secreted soluble hexamers. Although the Ns1 protein plays essential roles during early viral replication, when it is required for negative-sense RNA synthesis, its precise function remains poorly defined (Mackenzie et al., 1996; Alayli and Scholle, 2016). The Ns2A protein is a small, hydrophobic, multifunctional, membrane-associated protein involved in RNA replication, modulation of the host antiviral interferon response, and the assembly/secretion of viral particles (Leung et al., 2008). The Ns2B protein is a necessary cofactor for the activity of NS3, which is a large multifunctional protein that works as a serine protease, a 5′ RNA triphosphatase, a nucleoside triphosphatase, and a helicase (Brecher et al., 2013). The Ns4A protein is an integral membrane protein with a highly hydrophobic C-terminal region designated as the 2k fragment, which mediates membrane rearrangements essential for viral replication (Miller et al., 2007; McLean et al., 2011). The Ns4B protein is another highly hydrophobic membrane-associated protein that has been reported to interfere with Signal transducer and activator of transcription 1 (STAT1) phosphorylation and block of the Interferon alfa and beta (IFN-α/β) signal transduction cascade (Munoz-Jordan et al., 2003). The Ns4B protein has been reported to be a negative modulator of the Ns3 helicase activity (Umareddy et al., 2006). The Ns5 protein is the largest flaviviral protein and exhibits multiple enzymatic activities, including RNA-dependent RNA polymerase (Ackermann and Padmanabhan, 2001), N-7 guanine and 2’-O ribose methyltransferase (Dong et al., 2012), and RNA guanylyl-transferase activities (Issur et al., 2009). Thus, these nonstructural proteins play essential roles during viral morphogenesis, being required for viral RNA production, modulation of the host immune response, and assembly of new viral particles.

## Antibody Response to Flaviviruses

During viral infections, the development of neutralizing antibodies can be crucial for host protection. In the case of flavivirus infection, the humoral response is essential to control viral replication and dissemination, and is mostly mediated by neutralizing antibodies directed against viral surface glycoproteins (Slon Campos et al., 2018). In addition, antibodies produced during flavivirus infection may also develop protective Fc-mediated roles, including complement fixation, antibody-mediated cellular cytotoxicity, and opsonization (Vogt et al., 2011). The main targets of the humoral adaptive immune response to flaviviruses are epitopes localized on the envelope, namely the prM and Ns1 proteins; however, antibodies targeting flavivirus epitopes are not limited to these proteins, as evidence suggests that they target all flavivirus structural and nonstructural proteins (Anandarao et al., 2005; Pierson and Diamond, 2008; Kuivanen et al., 2014; Versiani et al., 2019). The envelope protein is considered the most relevant B-cell antigen target, conferring protective immunity by eliciting neutralizing antibodies; however, in the case of some flaviviruses, antibodies directed to the envelope protein can increase the infection efficiency under certain conditions by inducing subneutralizing antibodies (Rey et al., 2018). Most known neutralizing antibodies recognize conformational epitopes on the virion surface, but linear and continuous amino acid sequences from antigenic determinants on viral proteins are able to induce antibodies with neutralizing activities. However, a detailed map and characterization of flavivirus linear immunodominant peptides remain to be elucidated.

Identification of the antibodies that recognize an epitope is essential to understand the humoral immune response against a specific antigen. In general, epitopes are divided into two main categories: linear epitopes, which are composed of a stretch of continuous and sequential amino acids that interact with antibodies based on their primary structure; and conformational epitopes, in which key amino acids are brought together by the folded protein to form more complex structures that participate in antibody binding (Sela et al., 1967). All immune reactive epitopes share the common need for discovery; however, linear B-cell epitopes may represent a more convenient alternative for identification and prediction. Although a reduced fraction of antibodies are directed to linear epitopes, they often demonstrate a higher binding affinity than antibodies directed to discontinuous epitopes (Jemmerson, 1987; Van Regenmortel, 1996). Furthermore, working with linear peptides may represent a more reproducible and convenient alternative during immune assays, mostly due to its reduced risk of allosteric changes, in contrast to conformational epitopes, in which protein production or preparation may directly affect epitope arrangement. There is an increasing amount of data regarding linear continuous immunoreactive peptides from flaviviruses; thus, a careful study of the immune epitope landscape may help to identify specific vaccine candidates, elucidate important immunological or immunopathological mechanisms related to natural infections or active immunization, and provide trustful data during immunosurveillance.

In this review, we summarize the findings regarding human B-cell linear reactive antigen peptides from flaviviruses reported in studies published between 1989 and 2020. In surveying the literature, we searched for continuous linear peptides that reacted with sera from naturally infected humans. In addition, peptides were required to show relevant immunological antibody reactivity (Immunoglobulin G (IgG) or M (IgM)), such as viral- or group-specific reactivity, a protective or immunopathological outcome, immunological or immunodiagnostic potential, and reactivity with monoclonal antibodies. Based on these criteria, we identified 153 peptides from structural and nonstructural proteins in the DENV (serotypes 1–4), ZIKV, WNV, JEV, and TBEV (**Table 1** and **Supplementary Table 1**). Unsurprisingly, the majority of the humoral immune reactive peptides were described for the DENV, predominantly from the DENV2 serotype, which is probably a reflection of its long-lasting impact on human health. Furthermore, we identified 7 peptides from DENV1, 24 from DENV3, 27 from ZIKV, 4 from WNV, 6 from JEV, and 4 from TBEV. Understanding the data on these linear immune reactive peptides may provide useful information for those investigating antibody interactions with the primary structure of epitopes, supporting seroepidemiological studies, vaccine target characterization, and the elucidation of immune-related mechanisms.

**Table 1:** Quantitative summary of all described peptides included in this review, indicating peptide origin region.

Described peptides									
Virus	Structural			Non-structural					
	Env	Cap	prM	Ns1	Ns2	Ns3	Ns4a	Ns4b	Ns5
DENV1	4	–	3	–	–	–	–	1	–
DENV2	39	8	3	25	1	–	4		–
DENV3	13	–	3	8		–	–		–
DENV4	–	–	–	–	–	–	–		–
ZIKV	6	–	5	15	1	–	–	–	–
JEV	1	4	–	1	–	–	–	–	–
WNV	2	–	2	–	–	–	–	–	–
TBEV	3	–	–	–	–	–	–	–	1
**Total**	**153 peptides**								

## Capsid Protein

**Figure 1:**
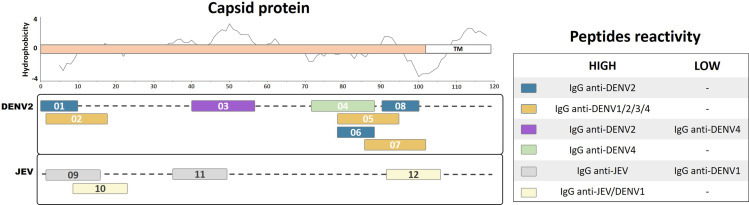
Linear representation of flavivirus capsid protein with representative topology and hydrophobicity prediction determined based on the ZIKV entry query of Uniprot Q32ZE1. Linear peptides are labeled by numbers and the antibody reactivity indicated by colors. The hydrophobicity prediction profile was determined using the amino acid sequence of the ZIKV (Genbank YP_002790881) with the Kyte & Doolittle hydropathy score scale (J. Mol. Biol. 157:105-132(1982). TM, Transmembrane region.

The flavivirus capsid protein is not exposed on the viral particle surface; therefore, a reduced level of B-cell stimulation is expected, with a consequently reduced number of identifiable immunogenic peptides (Kuhn et al., 2002; Anandarao et al., 2005). Antibodies against the capsid protein have been detected in the sera of DENV-infected patients in several studies (Huang et al., 1996; Anandarao et al., 2005; Nadugala et al., 2017). In this review, we summarize 12 linear immunogenic peptides from DENV2 and JEV capsids that were described during natural human infection (**Figure 1**). A linear epitope mapping study of the DENV2 capsid protein, based on predicted hydrophilic regions, identified three clusters of overlapping peptides (Anandarao et al., 2005): one cluster mapped to the N-terminal region, while two clusters mapped to the C-terminal region of the capsid protein. Three peptides showed the highest detection signals using DENV2 human IgG in the ELISA mapping assay (Pep01, MNNQRKKARN; Pep06, NVLRGFRKEI; and Pep08, MLNILNRRRR). However, none of these peptides developed detectable signals using DENV2 human IgM. Another peptide mapping study on the DENV2 capsid protein that used sera from DENV 1–4 infected patients detected five immunogenic peptides (Nadugala et al., 2017). Three of these peptides (Pep02, NNQRKKARNTPFNMLKR; Pep05, NVLRGFRKEIGRMLNIL; and Pep07, KEIGRMLNILNRRRRTA) showed elevated IgG recognition levels using sera from all of the DENV serotypes, suggesting that these peptides are highly antigenic and broadly reactive. One linear peptide (Pep04, IKKSKAINVLRGFRKEI) demonstrated elevated IgG recognition only for DENV4-infected patients. In addition, another peptide (Pep03, GRGPLKLFMALVAFLRFL) demonstrated elevated IgG recognition for DENV2-infected patients (100%) and partial detection of DENV4-infected patients (50%), suggesting that it is potentially a DENV2-specific epitope. Furthermore, an epitope mapping study of the JEV capsid protein identified two linear peptides (Pep09, TKKPGGPGKNRAINM; and Pep11, MSLLDGRGPVRFVLA) that specifically reacted with IgG from JEV-vaccinated children or seroconverted patients (Huang et al., 1996). However, they showed limited reactivity with IgG from DENV1 patients, suggesting that these peptides are specific immunodominant JEV epitopes. In contrast, two additional linear peptides from JEV (Pep10, GKNRAINMLKRGLPR; and Pep12, IDAVNKRGRKQNKRG) demonstrated broad reactivity with IgG from both JEV- and DENV1-infected patients, indicating that they may be potential group-specific epitopes.

## Pre-Membrane Protein

**Figure 2:**
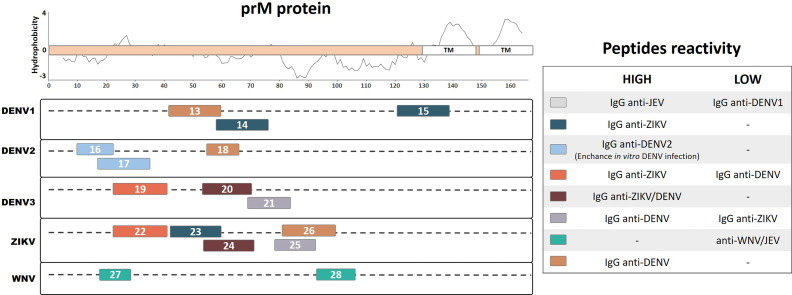
Linear representation of the flavivirus prM protein with representative topology and hydrophobicity prediction determined based on the ZIKV entry query of Uniprot Q32ZE1. Linear peptides are labeled by numbers and the antibody reactivity indicated by colors. The hydrophobicity prediction profile was determined using the amino acid sequence of the ZIKV (Genbank YP_002790881) with the Kyte & Doolittle hydropathy score scale (J. Mol. Biol. 157:105-132(1982). TM, Transmembrane region.

A significant subset of flavivirus antibodies is directed against the prM/M protein, which corresponds to a substantial proportion of memory B cells that are fully cross-reactive among the four DENV serotypes (de Alwis et al., 2011). Studies have demonstrated that antibodies against prM/M, even at high concentrations, have low *in vitro* and *in vivo* neutralization activities; in contrast, they potently promote antibody-dependent enhancement (ADE) of viral infection (Dejnirattisai et al., 2010; de Alwis et al., 2014). Interestingly, a previous study has demonstrated that human IgG antibodies against prM protein can distinguish between previous infection with the DENV and JEV (Cardosa et al., 2002). We identified 16 continuous prM linear peptides from DENV1, DENV2, DENV3, ZIKV, and WNV that demonstrated immunogenic characteristics during natural human infection (**Figure 2**). Using an ELISA mapping assay with recombinant linear peptides from the DENV1 and ZIKV prM region, a previous study identified one specific linear peptide from DENV1 (Pep13, ELCEDTMTYKCPRITEA) and one from ZIKV (Pep26, HKKGEARRSRRAVTLPSH) that developed higher detection signal levels with IgG from convalescent DENV patients, as compared with that from convalescent ZIKV patients. In contrast, two linear epitopes from DENV1 (Pep14, AEPDDVDCWCNATDTWV; and Pep15, QRVETWALRHPGFTVIAL) and one from ZIKV (Pep23, HMCDATMSYECPMLDEGV) were reported to develop higher detection signal levels with IgG from convalescent ZIKV patients than that from DENV patients (Kam et al., 2019). These results suggest that region 43–59 of the DENV1 and ZIKV prM proteins may act as a flavivirus-specific recognition peptide, given that Pep13 and Pep23 respectively were able to recognize the homologous IgG antibodies from convalescent DENV and ZIKV patients with higher detection signal levels. Meanwhile, the higher heterologous detection signals for Pep14, Pep15, and Pep26 from the prM region of DENV1 and ZIKV, as compared with those from the homologous antibodies, indicate that they may develop important roles during natural infection with flaviviruses. Interestingly, Pep15 from DENV1, which is highly reactive with ZIKV IgG antibodies, partially overlaps with the predicted transmembrane region of prM containing highly hydrophobic amino acids (**Figure 2**). Further studies are necessary to investigate the roles of antibodies against these linear peptides during homologous or heterologous infections.

A murine monoclonal antibody (mAb) that poorly neutralizes the DENV but potentially enhances its infectivity was characterized using a phage-display peptide library (Luo et al., 2013). This mAb demonstrated specific recognition of amino acids 14–18 of the DENV1-4 prM protein (Pep16, IVSRQEKGKS, with binding motif VS/GKTE) but not to other flaviviruses. This linear peptide from DENV2 was reported to be reactive based on ELISA detection with IgG from convalescent DENV2 patients. Furthermore, a prM protein mapping study identified a major immunodominant linear epitope from DENV2 (Pep18, KQNEPEDI) that recognizes IgG antibodies from DENV-infected convalescent patients with a higher signal than those from healthy individuals, making it a potential diagnostic peptide for the DENV (Song et al., 2013). Moreover, a prM linear peptide from DENV2 (Pep17, KGKSLLFKTEDGVNMC) was identified through peptide scanning and bioinformatic analysis (Luo et al., 2015). This peptide reacted with IgG from convalescent DENV2 patient sera and was found to enhance DENV infection *in vitro*. Collectively, Pep16, Pep17, and Pep18 recognize specific DENV IgG antibodies and may be important in relevant ways during DENV2 infection; however, we cannot overlook their potential role in cross-reactivity during heterologous infection with other DENV serotypes or with other flaviviruses.

A mapping study based on prM protein identified linear reactive peptides from DENV3 and ZIKV using IgG serum samples from convalescent DENV and ZIKV patients (Amrun et al., 2019). Among the reactive peptides, one linear peptide from DENV3 and one from the ZIKV (Pep20, HITEVEPEDIDCWCNLT; and Pep24, LDEGVEPDDVDCWCNTT) that reacted with both anti-ZIKV and anti-DENV antibodies were identified as potential common flavivirus-reactive antigens. The same study described a potential specific peptide from DENV3 (Pep21, TSTWVTYGTCNQAG) and a cross-reactive peptide from the ZIKV (Pep25, YGTCHHKKGEARRSR), which were detected at higher frequencies in IgG from DENV patients as compared with ZIKV patients. The study also identified a potential ZIKV-specific peptide (Pep22, SFPTTLGMNKCYIQIMDL) and a DENV3 cross-reactive peptide (Pep19, LFKTASGINMCTLIAMDL) on the prM region. These peptides reacted at higher rates with IgG from convalescent ZIKV patients compared with that from convalescent DENV patients. These results suggest that the DENV3 and ZIKV prM proteins both contain potential virus-specific linear peptides as well as potential cross-reactive linear peptides, which may provide relevant diagnostic tools and also develop important roles during flavivirus infection.

During WNV infection, most neutralizing antibodies are directed against the envelope protein; however, a subset of antibodies have been reported to additionally recognize the prM/M protein (Engle and Diamond, 2003). Two linear peptides from the WNV prM region (Pep27, VTDVITIPTA; and Pep28, SLTVQTHGESTLA) were predicted to be immunogenic B-cell peptide epitopes according to bioinformatic analyses (Gangwar et al., 2012). The authors then chemically synthesized these peptides and subjected them to ELISA using human sera that showed neutralizing activity against the WNV. Both peptides developed a low detection signal with anti-WNV and anti-JEV convalescent human serum samples, indicating the potential for cross-reactivity that may lead to further implications during infection. Although evidence suggests that antibodies against flavivirus prM proteins may cross-react among different viral species, a relatively low number of studies to date have investigated the potential cross-reactivity of linear epitopes. As such, at present, there are a very limited number of peptides from naturally infected humans that have been described.

## Envelope Protein

**Figure 3:**
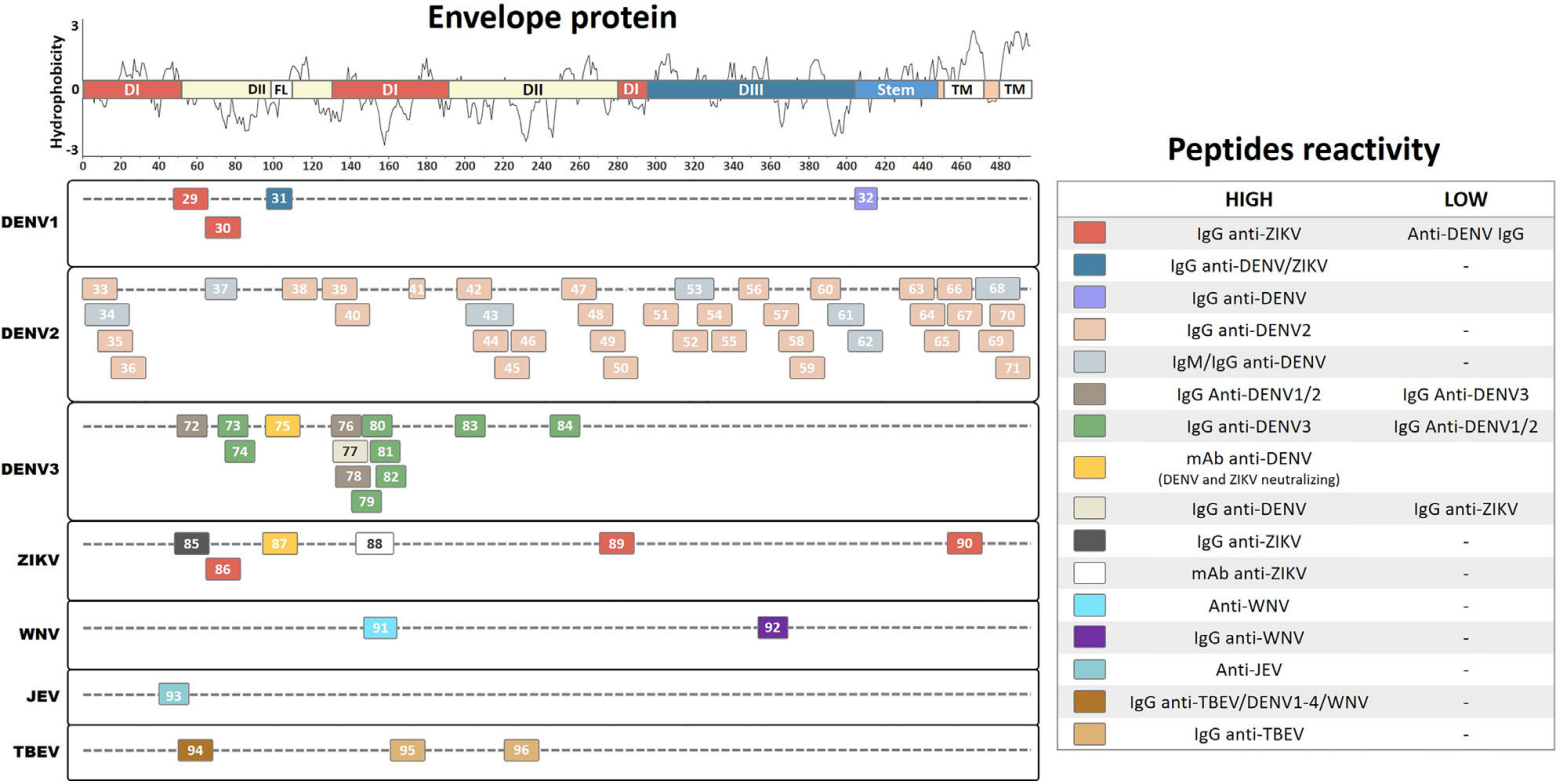
Linear representation of Flavivirus envelope protein. The representative topology and domain demarcation were determined based on the ZIKV entry query of Uniprot Q32ZE1. Linear peptides are labeled by numbers and the antibody reactivity indicated by colors. The hydrophobicity prediction profile was determined using the amino acid sequence of the ZIKV (Genbank YP_002790881) with the Kyte & Doolittle hydropathy score scale (J. Mol. Biol. 157:105-132(1982). DI/II/III, Domains I, II and III; FL, Fusion-loop; TM, Transmembrane region.

The vast majority of defined reactive antibodies to flaviviruses are directed against the envelope protein, a surface-exposed structure on the virus particle. Antibodies elicited against the envelope protein have critical roles during protection or ADE to flavivirus infection (Pierson et al., 2008; de Alwis et al., 2014). Therefore, it is essential to understand the antigenic determinants on the envelope protein of flaviviruses. Here, we summarize 68 linear B-cell immunogenic peptides previously identified and described in the literature for DENV1, DENV2, DENV3, ZIKV, WNV, JEV, and TBEV on the envelope protein (**Figure 3**).

A study of overlapping epitope mapping combined with ELISA screening for DENV1 and ZIKV envelope protein identified two linear peptides from DENV1 (Pep29, EVTNPAVLRKLCIEAKIS; and Pep30, ISNTTTDSRCPTQGEATL) that demonstrated higher levels of IgG in convalescent ZIKV patients than those from convalescent DENV patients (Kam et al., 2019). In addition, the same study demonstrated that a peptide region on the ZIKV envelope protein (Pep86, ISDMASDSRCPTQGEAYL) reacted with anti-ZIKV IgG and with at a reduced level with anti-DENV IgG antibodies, demonstrating the potential cross-reactive properties of this region (Kam et al., 2019). Furthermore, this study identified a peptide from the ZIKV envelope region (Pep85, TVSNMAEVRSYCYEASIS) that is specifically recognized by anti-ZIKV IgG and that fails to develop much of a detection signal with anti-DENV sera, suggesting that it could potentially be used to specifically diagnose the ZIKV.

A study using molecular dynamics simulations and energy decomposition predictions to analyze the DENV envelope protein identified a number of putative linear epitope sequences (Bergamaschi et al., 2019). One linear peptide was identified in the fusion loop region (Pep31, DRGWGNGCGLFG), which is responsible for establishing contacts with the host membrane during infection (Fritz et al., 2011). This peptide was then chemically synthesized and immobilized on a microarray for serological discrimination analysis. The results showed that it demonstrated elevated specificity and sensitivity to IgG from convalescent DENV patients compared with IgG from healthy individuals. Moreover, the peptide demonstrated comparable levels of cross-detection to IgG from convalescent ZIKV patients, probably due to the high level of sequence identity among the different flaviviruses. This suggests that the peptide may be a pan-flavivirus epitope with immunodiagnostic potential. Furthermore, another study predicted and evaluated a linear epitope from DIII of the envelope protein region of DENV1 (Pep32, RGARRMAIL), which is 89% conserved among the four DENV serotypes (Sanchez-Burgos et al., 2020). This study demonstrated that Pep32 reacted at a high frequency with IgG antibodies from DENV patients showing no warning symptoms or with mild disease during the early phase of infection; however, the peptide only showed a very limited detection frequency when IgG from patients with severe disease was used. This result suggests that reduced levels of antibodies against this peptide indicate a potential disease risk factor, and that the induction of antibody production against the peptide in vaccine formulations represents a beneficial alternative for host protection.

A study combining multistep sequence- and structure-based bioinformatic prediction with an overlapping synthetic peptide library of the entire DENV2 envelope protein identified and evaluated a number of potential linear B-cell epitopes (Ramanathan et al., 2016). Using a combination of epitope extraction and ELISA on purified IgG from DENV2-infected patients, the authors identified 29 linear peptides. Compared with the control groups, 8 peptides demonstrated reactivity only by ELISA (Pep51, KGMSYSMCTGKFKIVKEI; Pep54, RVQYEGDGSPCKIPFEIM; Pep55, GSPCKIPFEIMDLEKRHV; Pep58, VNIEAEPPFGDSYIIIGV; Pep59, PFGDSYIIIGVEPGQLKL; Pep69, SRSTSLSVSLVLVGVVTL; Pep70, VSLVLVGVVTLYLGAMVQ; and Pep71, SLVLVGVVTLYLGAMVQA), while 12 peptides demonstrated reactivity only by epitope extraction coupled with mass spectrometry (Pep33, MRCIGISNRDFVEGVSGG; Pep36, VSGGSWVDIVLEHGSCVT; Pep40, NLEYTIVITPHSGEEHAV; Pep44, KAWLVHRQWFLDLPLPWL; Pep45, LPWLPGADTQGSNWIQKE; Pep47, LGSQEGAMHTALTGATEI; Pep49, ATEIQMSSGNLLFTGHLK; Pep57, VTEKDSPVNIEAEPPFGD; Pep63, VFTSIGKALHQVFGAIYG; Pep64, ALHQVFGAIYGAAFSGVS; Pep66, SGVSWTMKILIGVIITWI; and Pep67, KILIGVIITWIGMNSRST). Nine peptides were detected by both techniques (Pep35, NRDFVEGVSGGSWVDIVL; Pep38, GLFGKGGIVTCAMFTCKK; Pep39, GKVVLPENLEYTIVITPH; Pep42, VLLQMEEKAWLVHRQWFL; Pep46, DTQGSNWIQKETLVNFKN; Pep48, MHTALTGATEIQMSSGNL; Pep50, SGNLLFTGHLKCRLRMDK; Pep52, VKEIAETQHGTIVIRVQY; and Pep65, AIYGAAFSGVSWTMKILI). Additionally, this study cosynthesized selected peptides (Pep38, Pep39, Pep42, Pep48, Pep52, and Pep65) together with a known T-helper epitope from the DENV and used it as a vaccine candidate in mice. In response to the vaccine, the mice developed a strong neutralization efficacy to DENV2 and also a weak cross-neutralization to DENV1 and DENV3. These findings provide novel insights into B-cell linear epitopes that could contribute toward future peptide-based vaccine development.

In addition, another study predicted and selected common peptides among all DENV serotypes showing no similarities with other flaviviruses from the envelope and Ns1 protein regions of DENV2. Subsequently, these peptides were chemically synthesized using a solid-phase synthesis technique and evaluated by ELISA to perform DENV-specific detection of IgM and IgG from the sera of infected patients (Nagar et al., 2020). This study identified seven continuous linear peptides from the envelope region (Pep34, RCIGISNRDFVEGVSGGSWVDIVL; Pep37, NTTTASRCPTQGEP; Pep43, MENKAWLVHRQWFLDLPLPWLPGADT; Pep53, KEIAETQHGTIVIRVQYEGDG; Pep61, WFKKGSSIGQMFETTMRGA; Pep62, RMAILGDTAWDFGSLGGV; and Pep68, MNSRSTSLSVSQVLVGIVTLYLGV) and four linear peptides from the Ns1 region (Pep102, TEQYKFQPESPSKLASAIQKA; Pep111, ALNDTWKIEKASF; Pep112, PETAECPNTNRAW; and Pep115, VLESEMVIPKNFAGPKSQ). These peptides demonstrated increased immunoreactivity toward acute and convalescent DENV2 patients compared with healthy controls, demonstrating elevated sensitivity and specificity levels for IgM (81%–97% and 82%–100%, respectively) and IgG (73%–94% and 82%–100%, respectively). Thus, this study described a number of important peptides that might be used as potential tools in diagnostic assays offering increased immunoreactivity, sensitivity, and specificity for DENV2 antibody detection. However, the possible cross-reactivity of these peptides to different DENV serotypes and to other flaviviruses still needs to be evaluated. Similarly, another study of epitope mapping identified a small linear peptide from the DI envelope protein of DENV2 (Pep41, EAELTGYG) that reacts with IgG from DENV2-infected patient sera (Aaskov et al., 1989). This study compared the detection of this peptide using sera from immunized rabbits to those from patients infected with DENV1–4; however, only a very limited number of human samples were used, which may have yielded an inaccurate picture of differences in antibody recognition. Collectively, these studies described a number of important peptides that might be useful for developing diagnostic assays with increased levels of immunoreactivity against DENV2 antibodies. However, evaluations of the potential cross-reactivity of these peptides to different DENV serotypes and to other flavivirus antibodies still need to be performed.

Another study described two linear peptides from the DENV2 envelope protein (Pep56, RHVLGRLITVNPIVT; and Pep 60, EPGQLKLNWFKKGSS) that are able to recognize IgG antibodies from convalescent DENV2 patients compared to those from healthy subjects (Li et al., 2011). These peptides were coupled with a T-cell epitope and used to immunize mice, leading to high levels of antibody production in the mice. These antibodies showed low neutralization activity against DENV2 and high *in vitro* lymphoproliferation, with a predominantly Th1-type immune response and a consequently reduced viremia *in vivo*. Thus, this study provides important insights into multi-epitope peptide-based strategies for vaccine development that are able to induce a long-lasting protective immune response.

An epitope mapping study of DENV3 synthesized 15-mer peptides with an overlap of 10 aa within the entire envelope protein, before evaluating their immunogenic reactivity by ELISA using DENV3 antibodies from convalescent patient sera (da Silva et al., 2009). The authors identified eight linear peptides (Pep73, DSRCPTQGEAVLPEE; Pep74, TQGEAVLPEEQDPNY; Pep79, ITVHTGDQHQVGNET; Pep80, GDQHQVGNETQGVTA; Pep81, VGNETQGVTAEITPQ; Pep82, QGVTAEITPQASTTE; Pep83, LLTMKNKAWMVHRQW; and Pep84: PEVVVLGSQEGAMHT) that are able to discriminate IgG from convalescent DENV3 patients compared with that from healthy controls. In addition, this study identified three linear peptides from the DENV3 envelope protein (Pep72, TQLATLRKLCIEGKI; Pep76, QYENLKYTVIITVHT; and Pep78, KYTVIITVHTGDQHQ) that develop a higher detection signal to IgG from DENV1 and DENV2 convalescent patients compared with that from convalescent DENV3 patients, making it possible to discriminate the DENV serotype of an infection. Interestingly, most of the specific DENV3 linear peptides were able to elicit IgG antibody production and induce IFN-γ secretion by CD4^+^ T cells from immunized BALB/c mice after peptide stimulation. Furthermore, another study performed epitope mapping of the DENV3 and ZIKV envelope proteins by ELISA-based screening with overlapping peptides using convalescent sera from ZIKV and DENV patients from Singapore and Thailand (Amrun et al., 2019). A potential DENV-specific peptide (Pep77, YENLKYTVIITVHTGDQH) and two potential ZIKV-specific peptides (Pep89, GALEAEMDGAKGRLSSGH; and Pep90: FKSLFGGMSWFSQILIGT) were identified in the envelope protein region that show higher IgG detection levels for sera from convalescent patients than for those from healthy controls. Collectively, these studies identified several immunodominant DENV3 and ZIKV peptides from convalescent patients that may be relevant for the specific diagnosis of these viruses and for vaccine development.

Another study performed ELISA epitope mapping of the prM, envelope, and Ns1 proteins from DENV3 and ZIKV to further characterize monoclonal antibodies isolated from secondary DENV-infected patients (Xu et al., 2012; Kam et al., 2017). Among the selected mAbs, the authors identified a neutralizing mAb that strongly recognizes highly conserved peptides from the fusion loop region, which is located on an exposed surface region on the distal face of the envelope proteins of DENV3 (Pep75, VDRGWGNGCGLFGKGSLV) and the ZIKV (Pep87, TLVDRGWGNGCGLFGKGS). This mAb did not develop *in vitro* ADE to ZIKV or DENV infection; instead, it demonstrated highly neutralizing activities. Similarly, a previous study investigating the immunogenicity of the ZIKV glycan loop identified a linear peptide from the envelope protein (Pep88, GSQHSGMIVNDTGHETDENRA) that is recognized by four other highly neutralizing mAbs from ZIKV-infected patients (Henderson et al., 2020). However, when mice were immunized with this glycan loop peptide alone, they did not develop a neutralizing antibody immune response against the ZIKV. This region contains a higher number of amino acids; therefore, it shows little homology compared with other flaviviruses and may act as a potential specific antigen. These results collectively indicate the existence of conserved and protective peptides in the fusion loop region, in addition to specific peptides on the glycan loop of the DENV3 and ZIKV envelope proteins that are recognized by naturally produced antibodies from infected individuals. These linear peptides could act as potential targets for immunotherapy, help to differentiate DENV from ZIKV infection, and contribute to vaccine development.

Similar to other flaviviruses, humoral immunity is essential for protective immunity against the WNV and JEV (Kurane, 2002; Diamond et al., 2003). Studies have shown that envelope protein antibodies are able to mediate a neutralizing and protective response against WNV and JEV infection (Chu et al., 2007; Gupta et al., 2015). One study used a bioinformatic approach to predict linear B-cell epitopes from the envelope proteins of the WNV and JEV (Gangwar et al., 2012). The authors identified two linear peptides (WNV Pep91: TTVESHGKIGATQAGRF and JEV Pep93: TLDVRMINIEASQLA) that demonstrated a strong virus-specific reactivity for mice and human sera without any cross-reactivity, representing potential diagnostic markers for differentiating between WNV and JEV infection. Furthermore, these peptides were used to develop a chimeric vaccine construct with a T-helper epitope that stimulated homologous *in vitro* neutralizing antibodies in mice, suggesting that these peptides could be potential targets for WNV and JEV vaccine development. Similarly, a different study used bioinformatics to predict B-cell epitopes in the WNV envelope protein and identified a 15-aa linear peptide from DIII of the envelope region (Pep92, LVTVNPFVSVATANS) (Herrmann et al., 2007). This peptide was cloned into a T7 bacteriophage vector and used in a phage display peptide-based ELISA to detect IgG from convalescent WNV patients, showing a detection limit titer of 1:51,200, a detection sensitivity of 67%, and a specificity of 100% compared with a commercial diagnosis kit. These results indicate that this approach could be used to prepare highly sensitive and specific anti-WNV immunoglobulin diagnostic kits that offer an easy antigen preparation strategy and increased stability.

Another study performed SPOT array epitope mapping using overlapping synthetic peptides on the full-length TBEV proteome (Kuivanen et al., 2014). The authors identified two immunodominant peptides in the envelope protein region (Pep95, ASFTVSSEKTILTMGEYG; and Pep96, LALPWKHEGAQNWNNAER) and one in the Ns5 protein region (Pep153, NTLTNIKVQLIRMMEGEG) that specifically react with IgG from naturally infected TBEV patients compared with that from TBEV-negative controls. These peptides did not show cross-reactive detection of DENV1–4 or WNV IgG from convalescent patients, indicating that they could be virus-specific antigen peptides. Interestingly, sera from TBEV-vaccinated individuals did not show elevated IgG reactivity against these peptides, suggesting that the peptides contain epitopes that are affected by conformational changes during viral inactivation in the vaccine formulation. In the same study, a peptide from the TBEV envelope region (Pep94, ENPAKTREYCLHAKLSDT) demonstrated broad reactivity with IgG antibodies from convalescent TBEV, DENV1–4, and WNV patients, indicating that it is a potential pan-flavivirus antigen peptide. In addition, a peptide from the DENV2 Ns2B region (Pep146, GLLVISGLFPVSIPITAA), which matches the Transmembrane (TM) region, demonstrated increased cross-reactivity with IgG from TBEV infected patients and reduced reactivity with that from DENV1–4 and WNV patients, indicating a potential effect on the immune response during cross-infection with DENV2 and the TBEV. However, this study used a limited number of clinical samples for epitope analysis, which may have affected the results.

## Ns1 Protein

**Figure 4:**
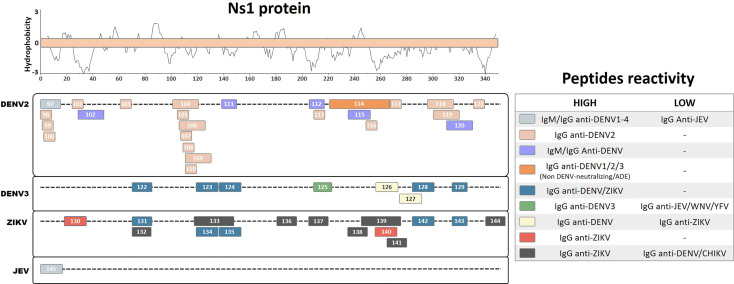
Linear representation of the NS1 protein. Linear peptides are labeled by numbers and the antibody reactivity indicated by colors. The hydrophobicity prediction profile was determined using the amino acid sequence of the ZIKV (Genbank YP_002790881) with the Kyte & Doolittle hydropathy score scale (J. Mol. Biol. 157:105-132(1982).

Among the nonstructural proteins, most immunoreactive peptides have been defined for the Ns1 protein, which is also a target of protective humoral and cellular responses (Lin et al., 1998; Timofeev et al., 1998; Brault et al., 2017). Antibody titers against the Ns1 protein are significantly higher in patients with Dengue hemorrhagic fever (DHF) than those with Dengue Fever (DF) and may target distinct regions of Ns1 protein, suggesting that it could be a marker of predicted disease severity (Jayathilaka et al., 2018). Furthermore, antibodies directed to Ns1 protein have been associated with an increased pathology mediated by autoreactive antibodies against self-endothelial proteins (Cheng et al., 2015). Thus, it becomes important to understand the roles of these antibodies and their epitopes during viral pathogenesis. In this review, we summarize results of 49 linear immunoreactive peptides for the Ns1 protein described for the DENV2, DENV3, ZIKV, and JEV (**Figure 4**).

A study of ELISA epitope mapping with synthetic peptides from predicted B-cell epitopes of the DENV2 Ns1 protein identified one linear peptide (Pep 97, DSGCVVSWKNKELKC) that specifically reacts with IgM and IgG antibodies from the sera of acute and convalescent dengue fever patients, but does not cross-react with antibodies from JEV patients (Huang et al., 1999). Additionally, this peptide reacts with antibodies from all four DENV serotypes as early as 2 days after the onset of symptoms. It also detects antibodies from primary and secondary infected patients with DF or Dengue shock syndrome (DSS). Furthermore, the corresponding peptide from the JEV (Pep145, DTGCAIDITRKEMRC) does not react with anti-JEV IgM but recognizes IgM from DENV2 patients, indicating a possible DENV-specific cross-reactive region with unreported potential pathological outcomes. Similarly, a different peptide ELISA mapping study of DENV2 antibodies from infected patients with DF and DSS identified strongly reactive peptides that interact with IgG from both murine and human convalescent DENV2 sera (Pep98, DSGCVVSWK; Pep99, GCVVSWKNK; Pep100, VVSWKNKEL; Pep101, VHTWTEQYK; Pep103, TRLENLMWK; Pep110, LRYSWKTWG; and Pep116, GPVSQHNNR) (Falconar, 2007). Similar reactivity was also observed with peptides with ELK/KLE-type motifs (Pep113, TWKIEKASF; Pep117, PWHLGKLEM; and Pep121, YGMEIRPLK), while higher IgG reactivity for DSS patients was observed with peptides containing an ELR motif (Pep105, RPQTELRY; Pep107, QPTELRYSW; and Pep108, TELRYSWKT), suggesting that these may be immunodominant serological markers.

Based on prediction algorithms from the DNASTAR, BCEPRED, and IEDB databases, one study selected eight potential diagnostic DENV2 peptides from the Ns1 protein that are common to all DENV serotypes (Nagar et al., 2020). These peptides were chemically synthesized, and their DENV antibody detection levels were evaluated. Four peptides (Pep102, TEQYKFQPESPSKLASAIQKA; Pep111, PETAECPNTNRAW; Pep112, ALNDTWKIEKASF; and Pep115, VLESEMVIPKNFAGPKSQ) were found to be the most immunoreactive, demonstrating elevated sensitivity and specificity in the detection of IgM and IgG from DENV patients compared with those from healthy controls. Furthermore, a different study performed an antigen microarray assay with overlapping peptides from DENV1–3 Ns1 proteins using sera from naturally DENV2-infected patients at the acute or convalescent phase, or at 12 months after the initial infection (Hertz et al., 2017). Two regions from Ns1 were detected with IgG antibodies from DF patients, convalescent DHF and DSS patients, and acute DSS patients, indicating their potential as serological disease markers (Pep104, KRSLRPQPTELKYSWKTWGK; Pep106, PQPTELKYSWKTWGKAKMLS; Pep109, LKYSWKTWGKAKMLSTESHN; Pep118, PSLRTTTASGKLITEWCCRS; and Pep119, TTASGKLITEWCCRSCTLPP). However, the antibody response against these peptides waned at 12 months after infection.

Another study characterized linear epitope recognition regions of nine monoclonal antibodies isolated from six acute or convalescent secondarily infected DENV2 patients by epitope mapping with truncated recombinant Ns1 proteins (Omokoko et al., 2014). All of these human monoclonal antibodies cross-reacted with a common epitope region from Ns1 of DENV1–3 but not with that from DENV4 (Pep114, KNCHWPKSHTLWSNGVLESEMIIPKNLAGPVSQHNYRPGYHTQITG), which has a highly conserved region in DENV1, 2, and 3 (WPKSHTLWSNGVLESEMIIPK). However, none of these mAbs developed neutralizing or ADE activities, which means that their potential physiological relevance during natural infection remains unclear.

Interestingly, another study identified human autoreactive antibodies during DENV infection, which reacted with human endothelial cells *in vitro*, potentially correlating with DHF syndrome development (Cheng et al., 2009). The authors identified a linear peptide from DENV2 Ns1 (Pep120, WCCRSCTLPPLRYRGEDGCW) by homologous sequence alignment with endothelial cell proteins. The autoreactive sera were further blocked by selective antibody depletion after incubation with this DENV2 peptide. Moreover, the same research group described a correlation between DHF patients and autoantibodies against self-proteins, which further correlates with the presence of IgM and IgG cross-reactive antibodies against the DENV2 peptide (Cheng et al., 2015).

A US patent registration described a linear peptide region on DENV3 Ns1 by ELISA epitope mapping using sera pooled from primary and secondary DENV3 patients during the peak of the anti-Ns1 antibody response (Marques et al., 2018). The authors identified an immunodominant linear peptide at position 209–233 (Pep125, SWKLEKASLIEVKTC) that is highly conserved in DENV3 but that varies considerably among other DENV serotypes and other flaviviruses. This peptide exclusively detected IgG from DENV-infected subjects, showing no cross-detection of IgG from human sera samples infected with other flaviviruses [WNV, JEV, and Yellow fever virus (YFV)]. Furthermore, the authors claimed that the antibody detection of this linear peptide region of Ns1 protein for all DENV serotypes and for other flaviviruses indicated the development of protective antibodies, which correlate with disease prognostics and the ability of the peptide to induce protective immunity against homologous flaviviruses. Moreover, the authors showed that antibody levels against this peptide region are inversely proportional to disease severity and may work as a protective factor against the development of DHF during secondary infections. However, the lack of expert revision during patent registration compromises the claims regarding the efficiency of these peptides, which need further investigation.

Another study performed ELISA epitope mapping and identified five peptides from the Ns1 protein of DENV3 (Pep122, QIANELNYILWENNIK; Pep123, GKAKIVTAETQNSSFIID; Pep124, GPNTPECPSASRAWNVWE; Pep128, TVVITENCGTRGPSLRTT; and Pep129, SCTLPPLRYMGE) and five peptides from the Ns1 protein of the ZIKV (Pep131, SVEGELNAILEENGVQ; Pep134, GKSYFVRAAKTNNSFVVD; Pep135, GDTLKECPLKHRAWNSFL; Pep142, KVHVEETCGTRGPSLRST; and Pep143, ECTMPPLSFRAK) as potential common linear flavivirus recognition peptides (Amrun et al., 2019). These peptides all developed similar detection results with IgG from convalescent ZIKV and DENV patient sera. In addition, two linear peptides from DENV3 (Pep126, RPGYHTQTAGPWHLGKLE; and Pep127, LDFNYCEGTTVVITENCG) were identified as potential DENV-specific peptides, demonstrating higher detection frequency with IgG from convalescent DENV patients compared with convalescent ZIKV patients. Similarly, an ELISA epitope mapping study of ZIKV- and DENV-infected patient sera from Brazil and Singapore identified two linear ZIKV peptides (Pep130, REGYRTQMKGPWHSEELE; and Pep140, TGVFVYNDVEAWRDRYKY) that specifically reacted with ZIKV IgG from convalescent patients, but not with IgG from DENV-infected patients, suggesting that these peptides could be serological immunodominant antigen markers (Kam et al., 2019). Interestingly, Pep140 was specifically recognized in ZIKV-infected pregnant women at higher recognition levels than those for other peptides, suggesting that this peptide is a common early marker of ZIKV infection in pregnant women.

Another epitope mapping study synthesized 33 linear peptides of 15–30 aa in length covering the whole ZIKV Ns1 protein and screened them for IgG detection using 20 ZIKV-positive convalescent sera samples (Lee et al., 2018). Eight linear peptides (Pep132, SVEGELNAILEENGV; Pep133, WGKSYFVRAAKTNNSFVVDGDTLKECPLKH; Pep136, PAVIGTAVKGKEAVH; Pep137, KNDTWRLKRAHLIEM; Pep138, IEESDLIIPKSLAGP; Pep139, SLAGPLSHHNTREGYRTQMKGPWHSEELEI; Pep141, GPWHSEELEIRFEEC; and Pep144, PESNLVRSMVTAGST) were found to develop strong positive reactivity compared with healthy control sera. Furthermore, these peptides did not show cross-reactive detection signals with IgG from DENV- and chikungunya virus (CHIKV)-infected patients. In particular, the peptides Pep132 and Pep133, which are located in the β-ladder surface region, demonstrated higher levels of IgG detection than those of the other peptides.

## Ns2B, Ns4A, and Ns4B

**Figure 5:**
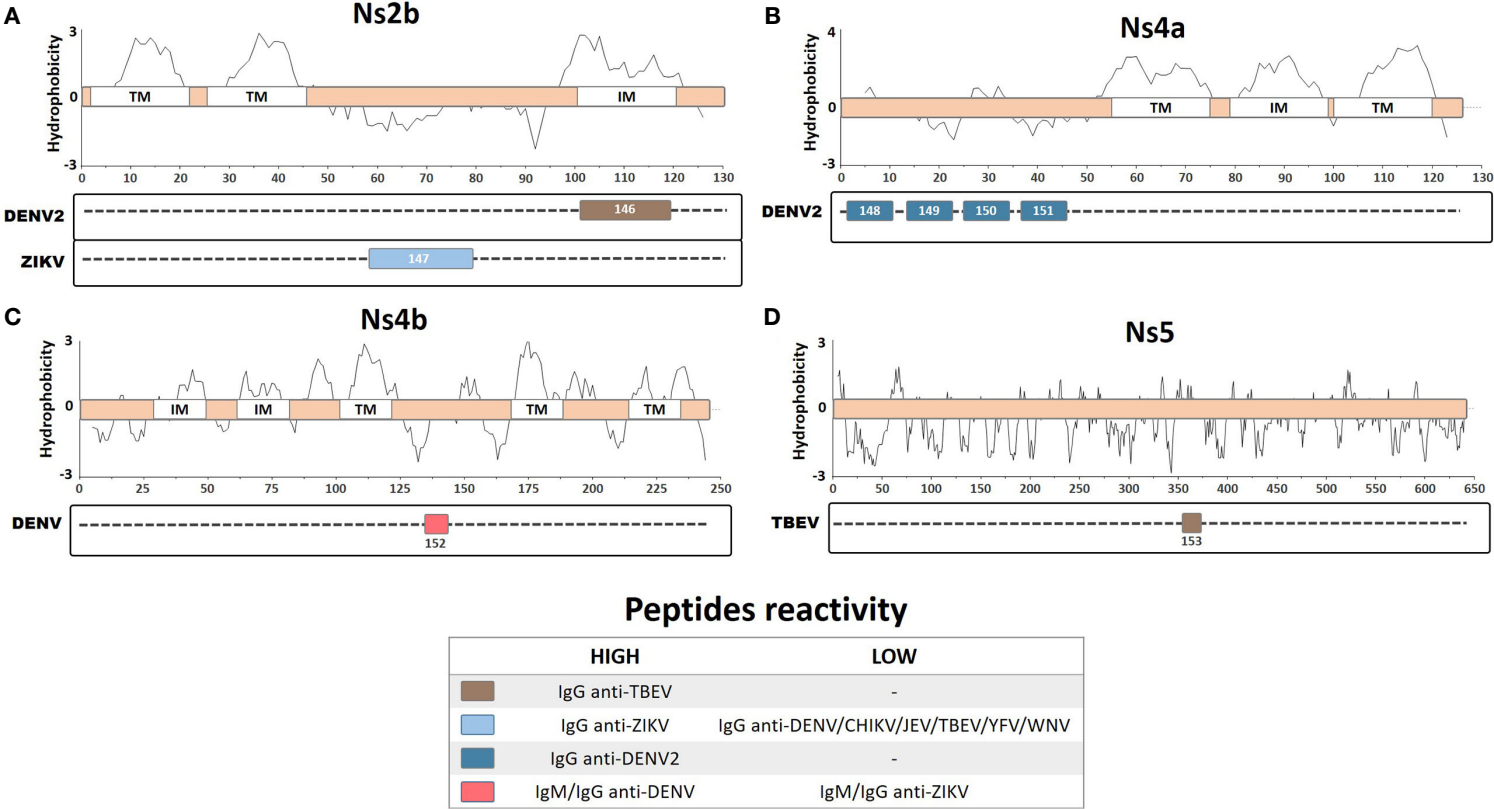
Linear representation of **(A)** Ns2b protein with topology based on the ZIKV entry query uniprot Q32ZE1, **(B)** Ns4a protein with topology based on the DENV2 entry query uniprot Q9WDA6, **(C)** Ns4b protein with topology based on the DENV4 query uniprot Q2YHF0, and **(D)** Ns5 protein. Linear peptides are labeled by numbers and the antibody reactivity indicated by colors. The hydrophobicity prediction profile was determined using the amino acid sequence of the ZIKV (Genbank YP_002790881), DENV2 (Genbank ACA48840), DENV4 (Genbank CH99664), and TBEV (Genbank ADQ00969.1), with the Kyte & Doolittle hydropathy score scale (J. Mol. Biol. 157:105-132(1982). TM, Transmembrane region; IM, Intramembrane region.

The antigenic potential of the nonstructural proteins is still not fully understood; thus, serological identification of antibodies directed against immunogenic peptides from these proteins may be intriguing. However, a number of studies have identified immunoreactive antibodies against these proteins, including elevated seroprevalence of anti-Ns4A during secondary infection, with Ns5 used as a specific antigen for serological detection (Se-Thoe et al., 1999; Wong et al., 2003; Narayan et al., 2016). Antibodies against Ns5 and other nonstructural proteins were observed during DENV infection, but their functional relevance is still unclear (Churdboonchart et al., 1991; Valdes et al., 2000). In this review, we included eight linear and continuous peptides described for the Ns2B, Ns4A, Ns4B, and Ns5 proteins from DENV, ZIKV, and TBEV (**Figures 5A–D**).

An epitope mapping study, using overlapping peptides from the ZIKV, DENV, YFV, WNV, Ilheus virus (ILHEV), Oropouche virus (OROV), and CHIKV, identified a specific linear peptide from ZIKV Ns2B protein (Pep147: DITWEKDAEVTGNSPRLDVALD) that is highly specific for ZIKV IgG detection (Mishra et al., 2018). This peptide demonstrated high sensitivity and specificity in detecting IgG from vaccinated or naturally infected patients, especially during early convalescence, as compared with IgG from other arboviruses. Thus, this peptide represents a potentially valuable diagnostic tool for performing serological evaluation and could be used for ZIKV surveillance to help manage exposed patients and to obtain insights into the epidemiological data regarding the ZIKV. A different study performed epitope mapping on predicted hydrophilic regions of the DENV2 Ns4A protein, which are likely to be more immunoreactive regions due to their exposure and accessibility (Anandarao et al., 2005). Four clusters of peptides in the Ns4A protein were identified, corresponding to the four most immunogenic peptides (Pep148, LTLNLITEMG; Pep149, PTFMTQKARD; Pep150, LDNLAVLHTA; and Pep151, GGRAYNHALS) that react with DENV2 IgG antibodies; however, none of these Ns4A peptides developed a detectable signal when tested with DENV2 IgM samples. Furthermore, an *in silico* study performed B-cell epitope mapping prediction on DENV proteins (excluding similar epitopes from YFV and ZIKV) and identified 9 DENV-conserved and 15 polymorphic epitopes (Versiani et al., 2019). These peptides were tested for reactivity using DENV-infected rabbit and patient sera. A linear peptide from the Ns4B region that was conserved in all DENV serotypes (Pep152, ATREAQKR) showed increased IgG and IgM detection by ELISA for all of the DENV serotypes from infected patients. However, the authors also observed limited cross-detection levels of IgG and IgM using ZIKV-infected patient sera, with 90% and 81% detection accuracy for DENV IgG and IgM, respectively, compared with a commercially available ELISA kit. Therefore, this linear peptide may represent an important diagnostic tool for differentiating DENV from ZIKV infection.

## Discussion

In this review, we evaluated data related to 153 linear immunoreactive peptides from viruses within the *Flavivirus* genus, including the DENV, ZIKV, JEV, WNV, and TBEV, as reported in 29 manuscripts published from 1986 to 2020. Unsurprisingly, the largest number of peptides in the literature was defined for the DENV, especially for the DENV serotype 2 (Narayan et al., 2016), followed by the ZIKV (Vogt et al., 2011), DENV3 (Dong et al., 2012), DENV1 (Filgueira and Lannes, 2019), JEV (Petersen et al., 2013), WNV (Pierson and Diamond, 2018), and TBEV (Pierson and Diamond, 2018). Consequently, the reduced number of peptides described for other flaviviruses may be partly due to their lesser impact on human health and the existence of effective vaccines or methods of prophylaxis (i.e., for the JEV, TBEV, WNV, and YFV). Regarding the described immunoreactive peptides, the majority were reported to react with antibodies from human convalescent serum samples. In terms of the antibody isotype, the studies reported total IgG reactivity most frequently, and in some cases, IgM reactivity was also evaluated, without providing any further information about antibody subclasses. This highlights the lack of information regarding the definition of reactive antibody classes, which may define effector immunological mechanisms involved in the immune response that are potentially associated with immune protection or involved in immunopathologies.

We observed that the reported immunoreactive peptides have been described across the whole flavivirus polyprotein, including the structural and nonstructural proteins; however, detailed and comprehensive data regarding the complete epitope mapping of flavivirus proteins are still lacking. The most comprehensive mapping with the greatest number of identified linear B-cell immunoreactive peptides has been completed for the envelope protein (68 peptides), followed by the Ns1 protein (49 peptides). These proteins are considered to be the most immunogenic targets of the immune system and are therefore the main targets for antiviral drug research, biological marker identification, immunodiagnosis, vaccine candidate development, and studies of pathogenesis (Zakaria et al., 2018; Chong et al., 2019; Araujo et al., 2020). Fewer immunoreactive peptides have been described for the capsid (Byk and Gamarnik, 2016) and prM (Mackenzie et al., 1996) proteins; however, important immunoreactive peptides have still been described for these proteins. These may serve as species-specific or group-specific serological diagnostic markers, may develop potential roles in ADE induction during DENV infection, and develop relevant roles during viral pathogenesis in secondary infection *via* cross-reactive antibodies. Furthermore, fewer immunoreactive peptides were also reported for the other nonstructural proteins, namely Ns2B (D.J. et al., 2007), Ns4A (Pierson and Diamond, 2018), Ns4B (Simmonds et al., 2017), and Ns5 (Simmonds et al., 2017), which develop very specific antibody reactivity to homologous viruses.

**Figure 6:**
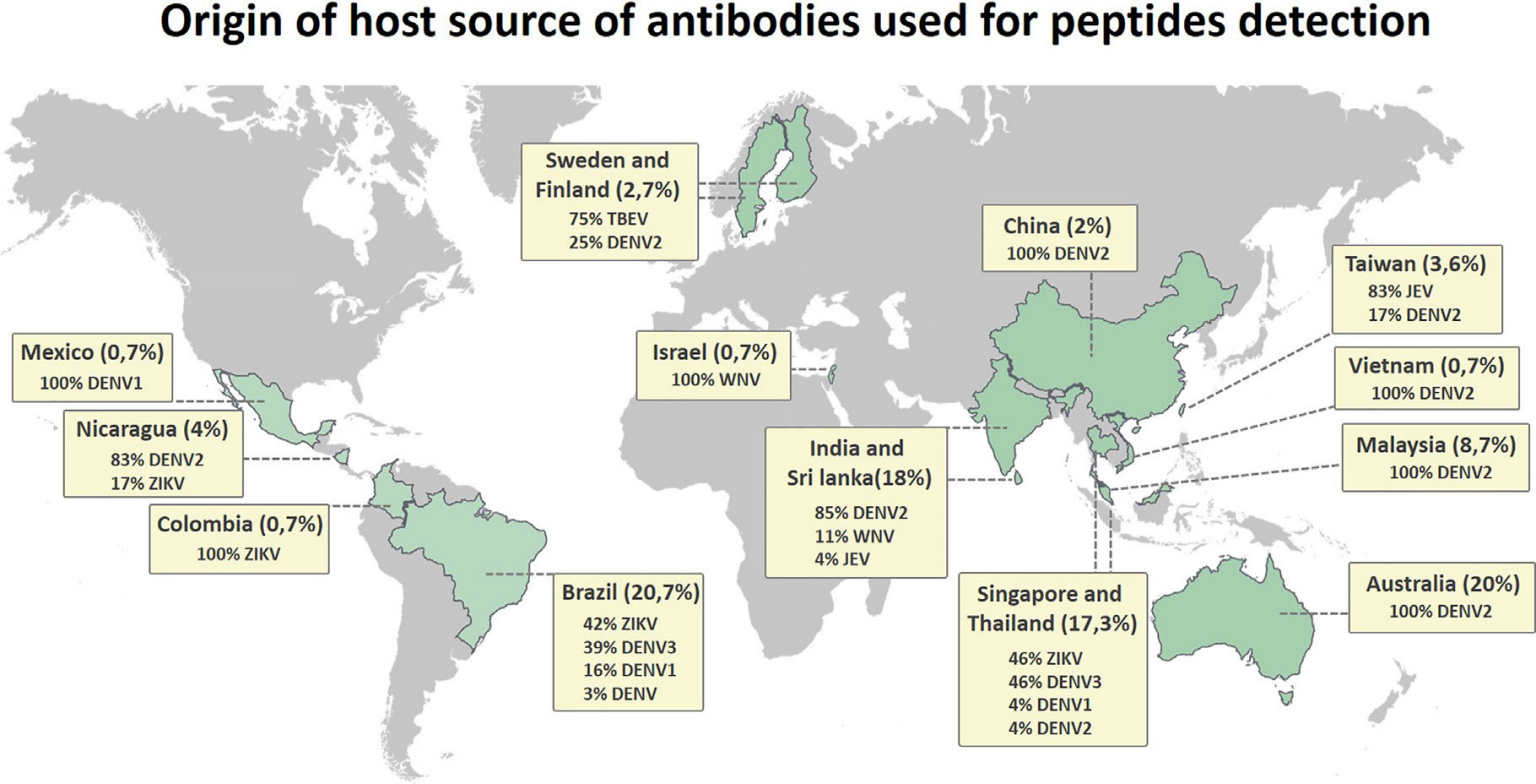
Distribution map showing the origin of the host source of antibodies used for detection of Flavivirus linear immunoreactive peptides, indicating the host region of origin and grouping the percentage of discovered peptides by country.

The flaviviruses consist of a complex group of antigenically related pathogens that are distributed all over the world, with insect vectors present in nearly every country. Considering that viral transmission may occur in many different populations at various times and places, the immune response and the consequent disease outcome may differ depending on the population. These processes can be affected by many uncontrolled variables, such as differences in host protein processing and presentation by the immune system, human leukocyte antigen (HLA) restrictions, and host genetic background. **Figure 6** shows the distribution of hosts from which the antibody repertoires were obtained for the immunogenic epitope mapping of peptides in this review. The vast majority of linear immune reactive peptides were described for sera from infected people distributed in Asia (India, Sri Lanka, China, Taiwan, Vietnam, Malaysia, Singapore, and Thailand), Latin America (Mexico, Nicaragua, Colombia, and Brazil), and Australia. It is not surprising that the vast majority and the most distributed identified peptides are for the DENV, which is present in almost all of the territories included in this review.

Flavivirus infection can cause a diverse range of disease severity in humans, ranging from mild febrile to severe neuroinvasive or hemorrhagic syndromes that last from days to months. During infection, the humoral immune response produces a variety of biological markers that indicate the possible prognosis of disease severity, including antibodies that recognize specific viral antigens. In this review, we have described a range of linear immunoreactive peptides that may be useful for disease prognosis, such as Pep32 from DIII of the DENV1 envelope protein, which demonstrated a positive IgG detection signal for sera from patients with mild DENV disease. However, patients progressing to a more severe disease state demonstrated very limited IgG detection using this peptide; therefore, it could be used as a biological marker of disease severity for DENV infection (Sanchez-Burgos et al., 2020). Additionally, five peptides from two regions of the DENV2 Ns1 protein (Pep104, Pep106, Pep109, Pep118, and Pep119) were suggested to be biological markers of DF, DHF, and DSS (Hertz et al., 2017). Furthermore, three peptides with ELR motifs from DENV2 Ns1 (Pep105, Pep107, and Pep108) were found to show a higher reactivity with IgG from DSS patients, indicating that they could act as additional serological markers (Falconar, 2007). Similarly, Pep120 from DENV2 Ns1 was identified as a possible biological marker recognized by antibodies from DHF patients and that could indicate DHF disease progression (Cheng et al., 2009). Furthermore, antibody recognition of Pep125 from DENV3 Ns1 may be a marker of the development of a protective immune response against DENV infection, indicating reduced chances of DHF disease progression (Marques et al., 2018). In addition, Pep140 from ZIKV Ns1 was reported to be a marker that is selectively recognized by antibodies from ZIKV-infected pregnant women, making it a specific early marker of infection (Kam et al., 2019). These results collectively demonstrate that detailed epitope mapping of linear immune reactive peptides during disease progression may enable the identification of important biological markers. These markers can help us to understand related protective or pathological mechanisms and distinguish disease severity in patients, leading to an appropriate healthcare response.

Over the course of a flavivirus infection, antibodies produced against the virus may lead to host protection or to the development of immunopathologies, such as ADE for the DENV. Both scenarios have been observed for antibodies against linear and continuous immunoreactive peptides. Interestingly, peptides such as Pep56 and Pep60 from the envelope protein have been associated with the development of *in vivo* protection against DENV2 infection due to a cellular immune response, although they induce low titers of neutralizing antibodies (Li et al., 2011). Furthermore, highly neutralizing ZIKV mAbs were reported to recognize Pep88 from the envelope protein; however, this peptide was not able to induce neutralizing antibodies when administered *in vivo* (Henderson et al., 2020). Pep91 and Pep93 from the WNV and JEV envelope proteins were found to stimulate viral-specific *in vivo* protection and production of neutralizing antibodies against homologous viruses, representing potential vaccine target candidates (Gangwar et al., 2012). Successful vaccine strategies have existed for decades for some flaviviruses, but some viral species remain without efficient candidates due to immunobiological complexities. Characteristics such as the induction of neutralizing antibodies by linear peptides may be desired during vaccine development, especially for those viruses that lack efficient immunization candidates or methods of prophylaxis. Unfortunately, the lack of appropriate animal models for some flaviviruses, due to the relatively high resistance to infection, still presents a problem in terms of evaluating vaccine immunization and protective immunity.

The identification of cross-reactive peptides from the *Flavivirus* genus has become an important subject of study, since B-cell epitopes may mediate immunopathologies associated with antibody cross-recognition and increase disease severity. For example, ADE during DENV infection is the result of poorly neutralizing antibodies that mediate an increased uptake of virus by Fcγ-bearing cells during secondary DENV infection (Littaua et al., 1990). A number of linear immunoreactive peptides may be recognized by cross-reactive antibodies and develop unexpected immunological outcomes, such as cross-protection or ADE, or may act as group-specific serological diagnostic markers. For example, Pep02, Pep03, Pep05, and Pep07 from the DENV capsid protein show elevated recognition levels by antibodies from all of the DENV serotypes (Nadugala et al., 2017). Pep10 and Pep12 from the JEV capsid protein also show increased cross-detection with antibodies from JEV and DENV1 patients, probably by acting as group-specific antigens (Huang et al., 1996). Peptides from the prM protein (such as Pep14, Pep15, Pep19, Pep20, Pep24, and Pep26) and from the envelope protein (such as Pep29, Pep30, and Pep86 of the DENV and ZIKV) also exhibit cross-detection with antibodies and may act as pan-flavivirus antigens (Amrun et al., 2019; Kam et al., 2019). Similarly, two linear peptides from the prM protein of the WNV (Pep27 and Pep28) were found to show similar cross-detection signals with anti-WNV and anti-JEV from convalescent patient sera (Gangwar et al., 2012). Furthermore, two linear peptides from the TBEV (Pep94 from the envelope protein and Pep146 from the Ns2B protein) demonstrated a broad reactivity with IgG antibodies from TBEV, DENV1–4, and WNV patients (Kuivanen et al., 2014). Interestingly, a mAb that recognized Pep16 from the DENV2 prM protein poorly neutralized DENV infection but potently enhanced *in vitro* infection (Luo et al., 2013). Moreover, Pep17 from the DENV2 prM was found to react with IgG from convalescent DENV2 patients and also demonstrated ADE to the DENV *in vitro* (Luo et al., 2015). Collectively, these data demonstrate that characterizing cross-reactive viral epitopes has become very relevant for developing vaccines and diagnostic tools. Thus, it is critical to better understand the landscape of immunoreactive peptides across the different flaviviruses. Indeed, the development of efficacious vaccines may help to ensure that specific antibodies are induced, while considering the potential for cross-reactive disease enhancement or further immunopathologies. Furthermore, the identification of specific viral antigens may contribute to the development of more precise diagnostic methods that improve our ability to diagnose and distinguish infections by different flaviviruses. This could further contribute to more reliable epidemiological surveillance, as well as to the development of specific clinical diagnoses leading to better patient healthcare management.

The current review aimed to summarize all of the linear immune reactive peptides from flaviviruses that have been reported to show relevant immune reactivity with antibodies from naturally infected humans. The current data are still limited, and more information regarding the immunoreactive landscape of flavivirus linear peptides still needs to be obtained. These data may be of great value to those involved in *Flavivirus* research, especially those interested in flavivirus antibodies. They could contribute to research in a wide range of areas, including the development of specific immunodiagnostic assays, the development of vaccines or therapeutic candidates, and the study of immunopathologies and viral pathogenesis.

## Author Contributions

MF collected and analyzed the data, designed the figures, and wrote the first version of the manuscript. LF supervised and provided the necessary resources. VA designed the main idea and supervised and critically reviewed the manuscript. All authors contributed to the article and approved the submitted version.

## Funding

This research was supported by Fundação de Amparo à Pesquisa do Estado de São Paulo (FAPESP) Scholarship number 2018/09383-3 and grant numbers 2019/27333-6 and 2019/26119-0.

## Conflict of Interest

The authors declare that the research was conducted in the absence of any commercial or financial relationships that could be construed as a potential conflict of interest.

## Publisher’s Note

All claims expressed in this article are solely those of the authors and do not necessarily represent those of their affiliated organizations, or those of the publisher, the editors and the reviewers. Any product that may be evaluated in this article, or claim that may be made by its manufacturer, is not guaranteed or endorsed by the publisher.
